# Behavioral Determinants of Patients' Willingness to Undergo Dental Implant Therapy: A Health Belief Model-Based Cross-Sectional Study

**DOI:** 10.7759/cureus.101742

**Published:** 2026-01-17

**Authors:** Nihalani Tanishq Shyamkumar, Rohit Patil, Shreya Bhukal, Neha Mukhopadhyay, Tanvi Bhardwaj, Seema Gupta

**Affiliations:** 1 Department of Oral and Maxillofacial Surgery, Maharana Pratap College of Dentistry and Research Centre, Gwalior, IND; 2 Department of Prosthodontics, Jawahar Medical Foundation's Annasaheb Chudaman Patil Memorial Dental College, Dhule, IND; 3 Department of Cardiology, All India Institute of Medical Sciences, New Delhi, New Delhi, IND; 4 Department of Dentistry, KPC Medical College and Hospital, Kolkata, IND; 5 Department of Public Health Dentistry, Sri Sukhmani Dental College and Hospital, Dera Bassi, IND; 6 Department of Orthodontics, Kothiwal Dental College and Research Centre, Moradabad, IND

**Keywords:** behavior, cost, dental implants, determinant, health belief models

## Abstract

Introduction: Dental implants represent the gold standard for tooth replacement; however, patient acceptance remains variable despite their proven clinical efficacy. This study aimed to investigate the sociodemographic and behavioral determinants influencing patients' willingness to undergo dental implant therapy using the health belief model (HBM) framework and to identify independent predictors of acceptance among adults with missing teeth.

Materials and methods: A cross-sectional analytical study was conducted over six months in 300 eligible adults (aged 18-65 years) with at least one missing tooth who were recruited using consecutive sampling. Data were collected using a structured self-administered questionnaire comprising sociodemographic details, single-item measures for eight HBM constructs on a five-point Likert scale, and a binary outcome variable (yes/no) for willingness to accept implant therapy. The data were then subjected to statistical analysis.

Results: Of the 300 participants, 155 (51.7%) expressed willingness to undergo implant therapy. Willingness was significantly associated with higher education and higher monthly income (both p = 0.001). Participants willing to opt for implants demonstrated significantly higher levels of perceived susceptibility, perceived severity, perceived benefits, self-efficacy, cues to action, and more favorable cost perception, along with lower dental anxiety (all p ≤ 0.001, except for cost perception: p = 0.046). No significant differences were observed for perceived barriers (p = 0.256). Binary logistic regression identified perceived severity (odds ratio (OR) = 1.57, p = 0.005), self-efficacy (OR = 1.23, p = 0.002), and cues to action (OR = 1.22, p = 0.049) as independent predictors. Relative importance analysis confirmed self-efficacy as the most influential behavioral construct.

Conclusions: Acceptance of dental implant treatment is significantly shaped by socioeconomic status and key components of the HBM, with self-efficacy playing the most influential role. Patients are more likely to opt for implant therapy when they feel confident in their ability to undergo the procedure, understand its long-term oral health benefits, and receive clear guidance from dental professionals.

## Introduction

Dental implants have transformed the field of restorative dentistry, providing a dependable and enduring solution for edentulism, characterized by increased success rates and enhancements in functionality, esthetics, and quality of life related to oral health [1]. Since the establishment of osseointegration principles, implants have become the preferred option over conventional removable or fixed prostheses for many patients, providing superior stability, preserving adjacent teeth, and preventing bone resorption. However, patient acceptance and willingness to undergo implant therapy remain variable and are influenced by multifaceted factors beyond clinical indications [2,3].

The literature highlights that psychological barriers, such as dental anxiety, fear of surgery, and unrealistic expectations regarding treatment outcomes or longevity, often deter patients from seeking treatment. Socioeconomic determinants play a pivotal role; higher education, income levels, and awareness significantly correlate with greater willingness to pay and accept implants, while cost remains the primary barrier for lower-income groups [4,5]. Demographic variables, including age, sex, and prior experience with prostheses, further modulate decision-making, with younger, more educated women showing a higher preference [6,7]. Knowledge gaps, sourced mainly from dentists or the media, also affect perceptions, underscoring the need for targeted education to align expectations with realistic benefits and risks [7,8].

Despite these advancements, disparities in implant uptake persist globally, particularly in underserved populations, emphasizing the importance of understanding behavioral pathways to enhance patient-centered care. This model-based analytical approach addresses these gaps by integrating psychological, socioeconomic, and cognitive factors. This cross-sectional study aimed to investigate the behavioral determinants and predictive factors influencing patients' willingness to undergo dental implant therapy among adults with missing teeth at a tertiary dental teaching institution.

The specific objectives were as follows: (i) to assess the levels of key health belief model (HBM) constructs among the study participants; (ii) to determine the associations between sociodemographic characteristics, HBM constructs, and patients' willingness to accept dental implants; (iii) to identify independent predictors of willingness to undergo dental implant therapy; and (iv) to derive evidence-based insights for developing targeted, patient-centered educational interventions aimed at addressing barriers and enhancing acceptance of dental implant treatment.

## Materials and methods

Study design

This study employed a cross-sectional analytical research design and was conducted in the Department of Prosthodontics at Jawahar Medical Foundation's Annasaheb Chudaman Patil Memorial Dental College, Dhule, India, from March 2024 to August 2024. The model-based approach enabled an in-depth exploration of how cognitive, emotional, and perceived risk-benefit factors shape patient decision-making. The study was carried out over a six-month period in the outpatient clinic of this tertiary dental teaching institution.

Study population and sampling

The target population consisted of adults aged 18-65 years who presented to the dental outpatient department (OPD) during the study period. Individuals with at least one missing tooth who could read English or a regional language and provided informed consent were included. Dental professionals, individuals previously treated with dental implants, and individuals with psychiatric disorders that impair judgment were excluded to avoid bias.

The sample size was determined using a priori power analysis conducted using G*Power software (Version 3.1.9.2; Heinrich-Heine-Universität Düsseldorf, Düsseldorf, Germany). Based on an expected dental implant acceptance rate of 24%, as reported in a previous study [9], and with statistical parameters set at 80% power and a 5% alpha error level, a minimum of 245 participants was estimated. To account for potential non-responses, this number was increased by 20%, resulting in a final sample size of 300 participants.

Study instrument: questionnaire development

Data were collected using a structured self-administered questionnaire developed based on the HBM constructs (see Appendices) [10]. Each construct was assessed using a single targeted item measured on a five-point Likert scale (1 = strongly disagree to 5 = strongly agree) [11]. The Likert scale is a freely available tool in the public domain. The questionnaire consisted of three sections: (i) demographic details, (ii) eight HBM-based behavioral construct items, and (iii) the outcome variable assessing willingness to undergo dental implant therapy (yes/no). The questionnaire was prepared in English and translated into regional languages to enhance comprehension and accessibility.

Validity and reliability of the questionnaire

Content validity was established through evaluation by a panel of five experts in prosthodontics, periodontology, public health dentistry, and behavioral sciences. The items were reviewed for conceptual relevance, clarity, and cultural appropriateness. The content validity index (CVI) for the instrument was 0.89, indicating excellent content adequacy of the instrument.

Questionnaire translation and pilot testing

The questionnaire was translated into a regional language using a standardized forward-backward translation procedure. An individual proficient in two languages initially translated the English document into another language, followed by a subsequent independent translator who conducted a back-translation into English. Any inconsistencies were resolved through consensus evaluation. A pilot test involving 30 adults was conducted to verify the clarity, readability, and flow. Feedback from the pilot resulted in minor wording adjustments, and the data from the pilot phase were excluded from the final analysis. Hard copies of the questionnaire were distributed to participants in the dental OPD, and a trained research assistant supervised the completion to minimize missing data. All responses were anonymized, and the completion time averaged 5-7 minutes.

Use of demonstration models in participant orientation

A brief orientation was provided using standardized demonstration materials to improve participants’ understanding of dental implants. A Dentsply Sirona Implant Educational Demonstration Kit (Dentsply Sirona, Charlotte, USA) and sample implant components from the Straumann® Standard Plus Implant System (Straumann Holding AG, Basel, Switzerland) were used to visually explain implant structure and function. These models served solely as educational aids and were not used in any clinical procedures.

Ethical considerations

The research protocol was scrutinized and endorsed by the Institutional Ethics Committee of Jawahar Medical Foundation's Annasaheb Chudaman Patil Memorial Dental College (Reference No. JMFACPMDC/IEC/2024/SS18). Written informed consent was obtained from each participant after a comprehensive explanation of the study's objectives, assurance of anonymity, and voluntary participation. The participants were free to withdraw at any stage without consequences. Data confidentiality was strictly maintained.

Data collection procedure

Eligible participants were approached after completing their routine dental consultation. After obtaining consent, participants were provided with either a printed or digital questionnaire based on their preferences. Completed questionnaires were checked for completeness prior to their acceptance. For digital submissions, responses were automatically recorded in an encrypted Google Drive (Google LLC, Mountain View, USA) that was accessible only to the research team. Hard copies were securely stored in a locked cabinet. All data were coded and entered into a spreadsheet for analysis.

Statistical analysis

Data were analyzed using the IBM SPSS Statistics software (Version 23.0; IBM Corp., Armonk, USA) and additional software for relative importance analysis, where required. Descriptive statistics, including means and standard deviations for continuous variables, medians and interquartile ranges (IQRs) for behavioral construct scores, and frequencies and percentages for categorical variables, were used to summarize the participant characteristics and responses. The distribution of behavioral construct scores was assessed for normality using the Shapiro-Wilk test. Owing to non-normal distribution, medians with IQRs were reported, and group differences between participants willing (yes) and not willing (no) to undergo dental implant therapy were examined using the Mann-Whitney U test for behavioral constructs and chi-square tests for categorical demographic variables. Binary logistic regression was performed to identify significant independent predictors of willingness to undergo implant therapy, with results presented as odds ratios (ORs) with 95% confidence intervals (CIs). A relative importance analysis was conducted to determine the contribution of each behavioral construct to predicting implant acceptance. Statistical significance was set at p < 0.05.

## Results

A total of 300 participants were included in the study, of whom 155 (51.7%) expressed willingness to undergo dental implant therapy (yes group), and 145 (48.3%) did not (no group). Significant associations were observed between willingness and education level (p = 0.001) and monthly income (p = 0.001). None of the participants with below-metric education or a monthly income <20,000 INR opted for implants, whereas 90 (30%) participants with higher education and 95 (32%) participants earning >50,000 INR/month were willing to undergo implant therapy. No significant associations were found for sex (p = 0.311), occupation (p = 0.485), or dental visits in the previous year (p = 0.282) (Table 1).

**Table 1:** Demographic characteristics of the study population by willingness to undergo dental implant therapy. Data are presented as frequency (n) and percentage (%). *p = 0.001 denotes statistical significance using chi-square test.

Variables	Categories	No (N = 145)		Yes (N = 155)		Total (N = 300)		Chi-square statistic (χ²)	p-value
		n	%	n	%	n	%		
Sex	Male	70	23%	95	32%	165	55%	1.03	0.311
	Female	75	25%	60	20%	135	45%		
Education	Below metric	65	22%	0	0%	65	22%	19.35	0.001*
	Intermediate education	50	17%	65	22%	115	38%		
	High education	30	10%	90	30%	120	40%		
Occupation	Service	110	37%	105	35%	215	72%	0.49	0.485
	Non-service	35	12%	50	17%	85	28%		
Income (INR/month)	<20000	50	17%	0	0%	50	17%	14.47	0.001*
	>50000	40	13%	95	32%	135	45%		
	20000-50000	55	18%	60	20%	115	38%		
Previous dental visit in last one year	Yes	105	35%	130	43%	235	78%	1.16	0.282
	No	40	13%	25	8%	65	22%		

Participants who were willing to undergo implant therapy reported greater awareness of the consequences of tooth loss, stronger confidence in the effectiveness of implants, higher confidence in their ability to undergo the procedure, and greater influence of motivating factors related to treatment uptake. They also showed a more favorable outlook toward treatment costs compared to those unwilling to opt for implants, with these differences being statistically significant (p < 0.05). No significant difference was observed between the groups with respect to perceived treatment-related obstacles (Table 2).

**Table 2:** Comparison of health belief model (HBM) constructs between participants willing and not willing to undergo dental implant therapy. Data are presented as median and IQR. *p < 0.05 denotes statistical significance using the Mann-Whitney U test. IQR: interquartile range

Behavioral construct	No (N = 145)		Yes (N = 155)		U statistic	p-value
	Median	IQR	Median	IQR		
Q1. Perceived susceptibility	3	2	4	1	7812.5	0.001*
Q2. Perceived severity	3	2	4	1	5237.5	0.001*
Q3. Perceived benefit	4	1	4	1	6562.5	0.001*
Q4. Perceived barrier	4	1	4	1	12025	0.256
Q5. Self‑efficacy	2	1	4	1	3200	0.001*
Q6. Cues to action	3	2	4	1	8325	0.001*
Q7. Cost perception	4	3	4	1	12662.5	0.046*
Q8. Dental anxiety	4	1	3	1	13825	0.001*

Binary logistic regression analysis (Table 3) identified perceived severity (OR = 1.57, p = 0.005), self-efficacy (OR = 1.23, p = 0.002), and cues to action (OR = 1.22, p = 0.049) as significant independent predictors of willingness to undergo dental implant therapy.

**Table 3:** Binary logistic regression analysis of factors influencing willingness to undergo dental implant therapy. ORs were recalculated from the provided coefficients as exp(B) for accurate interpretation. Results are expressed as OR with 95% CI. *p < 0.05 denotes statistical significance. SE: standard error; OR: odds ratio; CI: confidence interval

Behavioral construct	Coefficient B	SE	Z statistic	p-value	OR	95% CI (lower limit-upper limit)
Q1. Perceived susceptibility	-0.35	0.42	0.82	0.414	0.71	0.31-1.62
Q2. Perceived severity	-0.56	0.55	2.93	0.005*	1.57	0.20-2.67
Q3. Perceived benefit	-0.51	0.56	0.91	0.365	0.60	0.20-1.81
Q5. Self‑efficacy	-1.48	0.48	3.10	0.002*	1.23	0.09-2.58
Q6. Cues to action	0.20	0.49	1.40	0.049*	1.22	0.46-3.20
Q8. Dental anxiety	-0.07	0.53	0.13	0.898	0.93	0.33-2.62

Table 4 shows that confidence in one’s ability to undergo implant treatment contributed the most to the likelihood of accepting implant therapy, followed by awareness of the seriousness of tooth loss and the presence of motivating influences encouraging treatment uptake. Other factors, including perceived obstacles, cost-related considerations, and emotional responses toward dental treatment, contributed minimally and did not show a statistically significant influence on dental anxiety.

**Table 4:** Relative importance of health belief model (HBM) constructs in predicting willingness to undergo dental implant therapy. Relative importance analysis was performed to determine the proportional contribution of each construct to the prediction of implant acceptance. *p < 0.05 denotes statistical significance.

Behavioral construct	Relative importance (%)	Raw score	Z statistic	p-value
Q1. Perceived susceptibility	9.01	0.033	1.06	0.289
Q2. Perceived severity	22.18	0.082	2.35	0.037*
Q3. Perceived benefit	12.99	0.048	1.31	0.190
Q4. Perceived barrier	-2.24	0.008	-0.57	0.571
Q5. Self-efficacy	47.61	0.177	3.59	0.021*
Q6. Cues to action	13.60	0.043	1.82	0.010*
Q7. Cost perception	0.35	0.001	0.20	0.838
Q8. Dental anxiety	2.02	0.008	0.74	0.454

## Discussion

This cross-sectional study explored the behavioral and sociodemographic determinants of willingness to accept dental implant therapy among adults with missing teeth. The results indicate that a greater willingness to opt for implant therapy was associated with higher educational attainment and income, stronger awareness of oral health risks, greater confidence in treatment outcomes and personal ability to undergo the procedure, increased motivating influences, and lower levels of dental anxiety. Perceived barriers showed no notable differences between the groups, whereas cost perception showed a marginal trend. The independent predictors included perceived severity, self-efficacy, and cues to action, with self-efficacy emerging as the most influential factor in the relative importance analysis. These results underscore the interplay of cognitive, emotional, and socioeconomic elements in treatment decision-making, aligning with prior research and offering nuanced insights into patient-centered-care.

The strong influence of socioeconomic factors on willingness echoes the established patterns in the dental implant literature. A previous study consistently identified low income as a primary deterrent to implant acceptance, often overshadowing the clinical benefits due to affordability concerns [12]. Similarly, higher education correlates with increased awareness and preference for advanced treatments, as more educated individuals are better equipped to comprehend long-term advantages such as improved function and esthetics [13]. For instance, research in diverse populations has shown that patients from lower socioeconomic strata exhibit reduced uptake, attributing this to financial constraints and limited access to information, which perpetuates disparities in oral health outcomes [12,13]. Our observations reinforce these findings, highlighting how socioeconomic gradients not only affect initial considerations but also exacerbate inequities in restorative dentistry, particularly in underserved regions, where implant therapy remains underutilized [14,15].

Psychological and cognitive factors, as framed by the HBM, further elucidate decision-making pathways [16]. The prominence of perceived severity and self-efficacy as predictors aligns with the core tenets of the HBM, where the recognition of health threats and confidence in managing interventions drive positive behaviors [16]. Previous applications of the HBM in dentistry, such as periodontal care and oral hygiene promotion, have demonstrated that enhancing perceived severity motivates adherence, while bolstering self-efficacy reduces procrastination in seeking treatment [16,17]. In the context of implants, studies have linked lower self-efficacy to heightened surgical fear, deterring patients despite the acknowledged benefits [18,19]. Cues to action, often from professional advice, also emerged as influential, consistent with evidence that dentist recommendations significantly influence patient choices regarding elective procedures. The lower dental anxiety among willing participants corroborates research identifying anxiety as a major psychological barrier, with fearful individuals avoiding invasive options such as implants because of anticipated pain or complications [19,20]. Notably, the lack of difference in perceived barriers suggests that while surgery-related concerns exist universally, they may not decisively tip the balance when outweighed by perceived gains, a nuance supported by willingness-to-pay analyses, in which benefits such as durability prevail over procedural drawbacks.

These insights have profound clinical implications in restorative dentistry. Clinicians should prioritize personalized counseling that amplifies the perceived severity of untreated tooth loss, such as risks to oral function and quality of life, while fostering self-efficacy through educational tools, such as demonstration models, as used in this study. Addressing dental anxiety using anxiety-reduction techniques, such as cognitive-behavioral strategies or sedation options, could enhance acceptance rates. Regarding socioeconomic barriers, integrating financial counseling, payment plans, or subsidies may democratize access, particularly in low-income groups, where cost perception borders on the prohibitive. A patient-centered approach incorporating HBM-based assessments during consultations can tailor interventions to individual profiles, ultimately improving treatment uptake and satisfaction. This aligns with the broader calls for person-centered care in implantology, emphasizing holistic evaluation beyond clinical metrics.

Notwithstanding these findings, certain limitations of this study should be acknowledged. The cross-sectional nature of the study restricts the ability to establish causal relationships, as attitudes toward implant therapy may change over time. The use of self-reported responses may be subject to reporting biases, including socially desirable answers, particularly for variables such as income and anxiety. Furthermore, the use of convenience sampling from a single center may limit the applicability of the results to other settings, such as rural populations or individuals who are not actively seeking dental treatment. Finally, although the HBM served as a comprehensive theoretical framework, the use of single-item assessments for each construct may not fully capture the complexity of participants’ perceptions.

Future research should pursue longitudinal designs to track the influence of HBM constructs on actual implant uptake over time. Interventional trials testing HBM-tailored education programs, such as multimedia modules targeting self-efficacy and anxiety, can evaluate their efficacy in diverse settings. Exploring cultural and regional variations in barriers, alongside economic modeling of cost-mitigation strategies, would further inform policies. Ultimately, integrating advanced behavioral theories with digital tools for personalized risk-benefit communication holds promise for bridging the gaps in implant acceptance.

## Conclusions

This study demonstrated that patients' willingness to accept dental implant therapy is influenced by both socioeconomic and psychological factors within the HBM framework. Higher education and income promote acceptance through greater awareness and affordability. Key drivers include the perceived severity of tooth loss, self-efficacy in undergoing surgery, and responsiveness to professional recommendations. However, perceived barriers and cost concerns did not decisively differentiate between the groups. Clinically, these findings emphasize the need for tailored counseling to enhance confidence, highlight long-term health risks, and address anxiety. By integrating educational tools and financial guidance, clinicians can improve uptake, reduce disparities, and foster equitable access to advanced restorative care.

## Appendices

Questionnaire

Note: The questionnaire was created by the authors of this study.

Section A: Demographic Information 1. Age: ____ 2. Gender: Male / Female / Other 3. Education Level: Below metric / Intermediate / Higher education 4. Occupation: Service / Non-Service 5. Monthly Income: <20,000 / 20,000-50,000 / >50,000 6. Previous Dental Visit in Last 1 Year: Yes / No 

Section B: Behavioral Constructs (5‑point Likert Scale) 1. I believe I am at risk of losing more teeth (Perceived Susceptibility). 1 = Strongly Disagree, 2 = Disagree, 3 = Neutral, 4 = Agree, 5 = Strongly Agree 

2. Missing teeth can severely impact oral health (Perceived Severity). 1 = Strongly Disagree, 2 = Disagree, 3 = Neutral, 4 = Agree, 5 = Strongly Agree 

3. Dental implants can improve my chewing efficiency (Perceived Benefit). 1 = Strongly Disagree, 2 = Disagree, 3 = Neutral, 4 = Agree, 5 = Strongly Agree 

4. I am concerned about the pain involved in implant surgery (Perceived Barrier). 1 = Strongly Disagree, 2 = Disagree, 3 = Neutral, 4 = Agree, 5 = Strongly Agree 

5. I feel confident about undergoing dental treatment if recommended (Self‑Efficacy). 1 = Strongly Disagree, 2 = Disagree, 3 = Neutral, 4 = Agree, 5 = Strongly Agree 

6. My dentist’s advice influences my treatment decisions (Cues to Action). 1 = Strongly Disagree, 2 = Disagree, 3 = Neutral, 4 = Agree, 5 = Strongly Agree 

7. I believe implants are too expensive (Cost Perception). 1 = Strongly Disagree, 2 = Disagree, 3 = Neutral, 4 = Agree, 5 = Strongly Agree 

8. I feel anxious when thinking about dental procedures (Dental Anxiety). 1 = Strongly Disagree, 2 = Disagree, 3 = Neutral, 4 = Agree, 5 = Strongly Agree 

Section C: Willingness to Undergo Implant Therapy 1. Are you willing to undergo dental implant treatment if needed? Yes / No / Not Sure 

## References

[1] Buser D, Sennerby L, De Bruyn H (2017). Modern implant dentistry based on osseointegration: 50 years of progress, current trends and open questions. Periodontol 2000.

[2] Sokolowski A, Huber S, Arefnia B, Pichler A, Lorenzoni M, Sokolowski A (2025). The influence of prosthetic treatments and implant-supported prostheses on posterior mandibular ridge atrophy: a retrospective cohort study. BMC Oral Health.

[3] Ghanem H, Afrashtehfar KI, Abi-Nader S, Tamimi F (2015). Impact of a ‘TED-style’ presentation on potential patients’ willingness to accept dental implant therapy: a one-group, pre-test post-test study. J Adv Prosthodont.

[4] Leung KCM, McGrath CPJ (2010). Willingness to pay for implant therapy: a study of patient preference. Clin Oral Implants Res.

[5] Re D, Ceci C, Cerutti F, Fabbro MD, Corbella S, Taschieri S (2017). Natural tooth preservation versus extraction and implant placement: patient preferences and analysis of the willingness to pay. Br Dent J.

[6] Nitschke I, Krüger K, Jockusch J (2024). Age-related knowledge deficit and attitudes towards oral implants: survey-based examination of the correlation between patient age and implant therapy awareness. BMC Oral Health.

[7] Rani S, Dhawan P, Kumar A, Kumar H, Tripathi R (2024). Knowledge, awareness and decision making of population visiting north indian institute towards dental implant as a treatment modality: a cross-sectional study. Int J Adolesc Med Health.

[8] Mayya A, D’Souza J, George AM, Shenoy K, Jodalli P, Mayya SS (2018). Knowledge and awareness of dental implants as a treatment choice in adult population in South India: a hospital-based study. Indian J Dent Res.

[9] Shaikh RN, Chauhan CJ, Prajapati N, Tintodana K, Patel G, Machchar A (2024). Knowledge, acceptance, and satisfaction about dental implant among patients of Patan district of Gujarat. J Pharm Bioallied Sci.

[10] Janz NK, Becker MH (1984). The health belief model: a decade later. Health Educ Q.

[11] Likert R (1932). A technique for the measurement of attitudes. Arch Psychol.

[12] Alamoush RA, Elmanaseer WR, Matar YW, Al-Omoush S, Satterthwaite JD (2022). Sociodemographic factors and implant consideration by patients attending removable prosthodontics clinics. Biomed Res Int.

[13] Kc Basnyat S, Sapkota B, Shrestha S, Rimal U (2020). Assessment of level of expectation and awareness towards dental implants among complete denture patients and partial denture prostheses wearers. Kathmandu Univ Med J (KUMJ).

[14] Choi J-S, Shin B-M, Park D-Y, Park G-Y, Choi Y-K (2022). The association between dental implant treatment experience and socioeconomic factors in Korean adults: a cross-sectional survey data analysis. Iran J Public Health.

[15] Abbas H, Aida J, Saito M (2019). Income or education, which has a stronger association with dental implant use in elderly people in Japan?: social inequalities in implant use. Int Dent J.

[16] Solhi M, Zadeh DS, Seraj B, Zadeh SF (2010). The application of the health belief model in oral health education. Iran J Public Health.

[17] Chan CCK, Chan AKY, Chu CH, Tsang YC (2023). Theory-based behavioral change interventions to improve periodontal health. Front Oral Health.

[18] Fatani B, Alrumayyan SF, Alsubaie RM, Alhussayen MS, Alharbi OA, Alsaleh RF, Al-Safadi A (2023). Factors affecting the choice of implant specialists among the Saudi population: a cross-sectional study. Cureus.

[19] Sağlanmak A, Arısan V (2024). Changes of dental implant surgery-related anxiety and pain with respect to ASA-physical status. J Clin Med.

[20] Weisensee W, Scheer M, Müller L (2012). Impact of anxiety parameters on prospective and experienced pain intensity in implant surgery. Implant Dent.

